# Location of Immunization and Interferon-γ Are Central to Induction of Salivary Gland Dysfunction in Ro60 Peptide Immunized Model of Sjögren's Syndrome

**DOI:** 10.1371/journal.pone.0018003

**Published:** 2011-03-28

**Authors:** Hongen Yin, Jelle L. Vosters, Nienke Roescher, Anil D'Souza, Biji T. Kurien, Paul P. Tak, John A. Chiorini

**Affiliations:** 1 Molecular Physiology and Therapeutics Branch, National Institute of Dental and Craniofacial Research, National Institutes of Health, Bethesda, Maryland, United States of America; 2 Division of Clinical Immunology and Rheumatology, Academic Medical Center, University of Amsterdam, Amsterdam, The Netherlands; 3 Department of Medicine, University of Oklahoma Health Sciences Center, Oklahoma City, Oklahoma, United States of America; Sheba Medical Center, Israel

## Abstract

**Introduction:**

Anti-Ro antibodies can be found in the serum of the majority of patients with Sjögren's syndrome (SS). Immunization with a 60-kDa Ro peptide has been shown to induce SS-like symptoms in mice. The aim of this study was to investigate factors involved in salivary gland (SG) dysfunction after immunization and to test whether the induction of SS could be improved.

**Methods:**

Ro60 peptide immunization was tested in Balb/c mice, multiple antigenic peptide (MAP)-Ro60 and Pertussis toxin (PTX) were tested in SJL/J mice. In addition, two injection sites were compared in these two strains: the abdominal area and the tailbase. Each group of mice was tested for a loss of SG function, SG lymphocytic infiltration, anti-Ro and anti-La antibody formation, and cytokine production in cultured cells or homogenized SG extracts.

**Results:**

Ro60 peptide immunization in the abdominal area of female Balb/c mice led to impaired SG function, which corresponded with increased Th1 cytokines (IFN-γ and IL-12) systemically and locally in the SG. Moreover, changing the immunization conditions to MAP-Ro60 in the abdominal area, and to lesser extend in the tailbase, also led to impaired SG function in SJL/J mice. As was seen in the Balb/c mice, increased IFN-γ in the SG draining lymph nodes accompanied the SG dysfunction. However, no correlation was observed with anti-MAP-Ro60 antibody titers, and there was no additional effect on disease onset or severity.

**Conclusions:**

Effective induction of salivary gland dysfunction after Ro60 peptide immunization depended on the site of injection. Disease induction was not affected by changing the immunization conditions. However, of interest is that the mechanism of action of Ro60 peptide immunization appears to involve an increase in Th1 cytokines, resulting in the induction of SG dysfunction.

## Introduction

Sjögren's syndrome (SS) is a systemic autoimmune disorder of unknown etiology. This autoimmune exocrinopathy is characterized by mononuclear cell infiltration in exocrine glands, principally the lachrymal and salivary glands (SGs). In serum, >75% of SS patients have autoantibodies against the nuclear antigens Ro (SSA) and La (SSB). These antibodies are associated with SS, but are not unique to the disease [1]. The pathogenic relevance of these autoantibodies is not clear and other autoantibodies involved in neuronal innervation, aquaporins, matrix metalloproteinases, and apoptosis have also been identified to be involved in the pathogenesis of SS (as reviewed in [2]).

To better understand the pathogenesis of the most common autoantibodies in SS, various animal models have been established by focusing on anti-Ro and La antibodies [3], [4]. Balb/c mice immunized with short Ro60 peptides developed anti-Ro and -La antibodies, SG lymphocytic infiltrates, and SG dysfunction, also seen in SS patients [5]. In addition, although the pathogenic function of autoantibodies against Ro and La is not clear, a number of previous studies imply that enhanced pro-inflammatory cytokines, such as interferon (IFN)-γ, interleukin (IL)-18 and IL-17, are highly related to increased anti-Ro antibody levels in SS [6]. This suggests a possible correlation between anti-Ro antibodies and autoimmune T cell mediated responses in SS. Moreover, previous studies from our own group have shown the role of T cell related cytokines (e.g. IFN-γ and IL-12) in salivary gland dysfunction [4], [7], [8]. Therefore, we have set up the same model to investigate the induction of SS-like symptoms, and the role of cytokines in SG dysfunction after Ro60 peptide immunization.

It was previously shown that only 30% of the immunized mice develop SG foci and variability in SG dysfunction was observed [5]. To further optimize this animal model, we tested other conditions for SS disease induction using the same peptide, and switched to SJL/J mice, a well established autoimmune prone animal model [9], [10]. We also added Pertussis toxin (PTX) to our peptide emulsion, which is an important additional adjuvant in experimental autoimmune uveoretinitis (EAU) in B10.A mice [11]. Moreover, based on our own experience (unpublished data, Roescher et al.) and on the literature [12], [13], higher antibody titers can be achieved by immunizing with a multiple antigenic peptide (MAP) instead of the conventional peptide. Therefore, we have immunized mice with MAP-Ro60 peptide and tested in two different injection sites for its effect on disease onset.

## Results

### Induction of anti-Ro60 antibodies and salivary gland dysfunction in Balb/c

**Figure 1:**
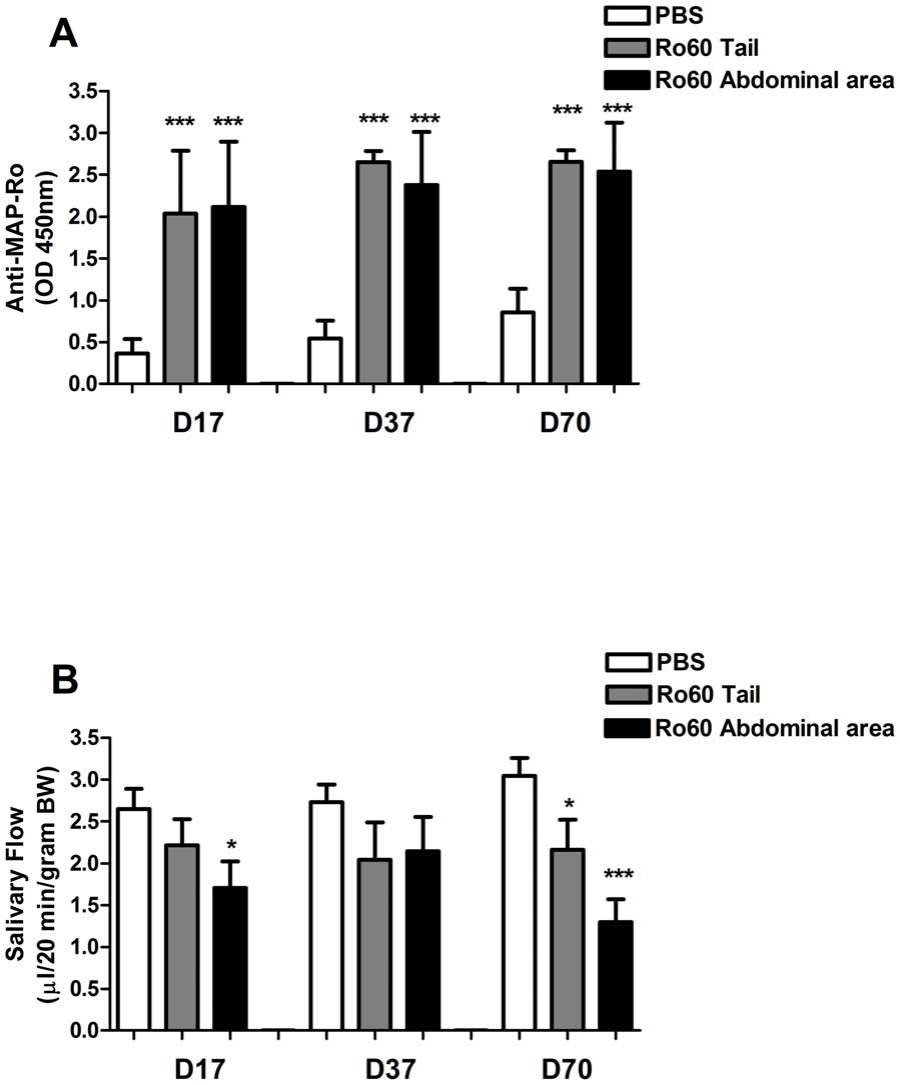
Increased anti-Ro60 antibodies and salivary gland dysfunction in Ro60-immunized Balb/c. Balb/c mice were immunized with Ro60 peptide or PBS (mock) in the tailbase or the abdominal area. Plasma from all animals were analyzed at the indicated time points for anti-MAP-Ro60 antibodies (**A**). The same mice were analyzed for pilocarpine stimulated salivary flow (**B**). Data shown are mean values +/- SEM for 10 mice/group. Significant differences are indicated (*** p<0.0001; * p<0.05) and were determined by unpaired Student's t-test. D: number of days post primary injection.

Balb/c mice were immunized with Ro60 peptide as previously described [5], and the induction of anti-Ro60 antibodies was assessed at day 17, 37 and 70. Both tailbase and abdominal area immunized Balb/c mice showed a significant increase in anti-MAP-Ro60 antibodies (p<0.0001) following the first boost, and this was sustained throughout the whole study (Figure 1A).

To investigate the effect of Ro60 peptide immunization on SG function, stimulated salivary flow was measured. Immunization with Ro60 peptide at the tailbase showed on day 70 a modest decrease in salivary flow rate (SFR) compared with controls (p = 0.04, Figure 1B). Changing the injection site to the abdominal area resulted in a more pronounced decrease in saliva production on day 70 (p<0.0001, Figure 1B). In addition, on day 17, a transient decrease in SFR following immunization in the abdominal area was observed (p = 0.02). These data confirm previous work showing that Ro60 peptide immunization in Balb/c mice can lead to a decrease in salivary flow. Furthermore, our comparison of immunization sites suggests that impaired SG function is affected by the site of injection.

### Salivary gland dysfunction is dependent on activation of Th1 cells

In order to understand the mechanism associated with the change in SFR following immunization in the abdominal area, as well as the direct role of the Ro60 peptide activated lymphocytes, cytokine expression was tested in cultures obtained from splenocytes re-stimulated with Ro60 peptide (Table 1). In the spleen, Ro60 specific cells from the abdominal area injected mice produced consistently higher levels of cytokines, e.g. IFN-γ, IL-12, and IL-10, compared with PBS or tailbase treated mice (Table 1). These data suggest a direct stimulation of Ro60 peptide on the splenic T cells when administered *in vivo*. In addition, the higher levels of cytokines in mice injected in the abdominal area further supports the abdominal area as the optimal delivery site for Ro60 antigen to the systemic immune system.

**Table 1:** Cytokine production after Ro60 peptide re-stimulated splenocytes from Balb/c mice.

		Th1-			Th17-	Th2-	
		IFN-γ	IL-12p40	IL-18	IL-17	IL-4	IL-10
	PBS	1.9	3.9	0.8	0.2	0.0	3.3
**Splenocytes**	Ro60-Tail	156.6	82.2	29.4	51.4	3.2	33.5
	Ro60-Abdominal area	313.9	146.7	32.8	36.6	2.7	42.2

Splenocytes were pooled from 10 mice/group. Culture supernatants were collected following incubation with or without (medium controls) Ro60 peptide and analyzed for levels of the indicated cytokines (in pg/mL). Cytokine levels from the medium controls were subtracted from the Ro60 peptide treated cells and the mean of each duplicate was used in this table.

We also analyzed cytokines in SG homogenates from tailbase or abdominal area immunized mice on day 95 post primary immunization. Data obtained from SG homogenates showed Th1 cell activation with elevated IFN-γ and IL-12, in abdominal area immunized mice compared with either PBS control or tailbase immunized mice (Table 2). This local effect on the SG further suggests a role for Th1 cytokines in the loss of SG activity in this model.

**Table 2:** Salivary gland cytokines in Ro60-immunized Balb/c mice.

		Th1-			Th17-	Th2-	
		IFN-γ	IL-12p40	IL-18	IL-17	IL-4	IL-10
	PBS	21.3	6.8	165.4	3.2	8.0	12.5
**SG homogenates**	Ro60-Tail	27.0	6.7	88.5	4.0	7.2	16.1
	Ro60-Abdominal area	50.8	13.5	172.1	6.6	10.3	14.3

Cytokine levels in the SG were determined after extraction of soluble protein from the SGs and correction for total protein concentration. Data from SG protein extracts are the mean (pg/mL) of 3 random samples from 10 mice/group for one experiment.

Previous research with other immunization models of autoimmune disease has suggested that the use of other strains of mice, different forms of peptide, or the use of secondary adjuvants can enhance disease onset and penetrance. Therefore, we tested disease induction in the autoimmune prone SJL/J mouse strain. This strain of mice has been shown to have salivary gland dysfunction following over expression of IL-12 in the thyroid gland [4]. In addition, we immunized with a MAP version of the Ro60 peptide, which is known to induce higher titers of antibodies [12], [13], and added a secondary adjuvant (PTX), which has been reported to be critical for disease induction in some strains of mice for experimental autoimmune uveoretinitis [11], and studied the effect on disease induction.

### SJL/J mice do not develop autoantibodies after MAP-Ro60 immunization

**Figure 2:**
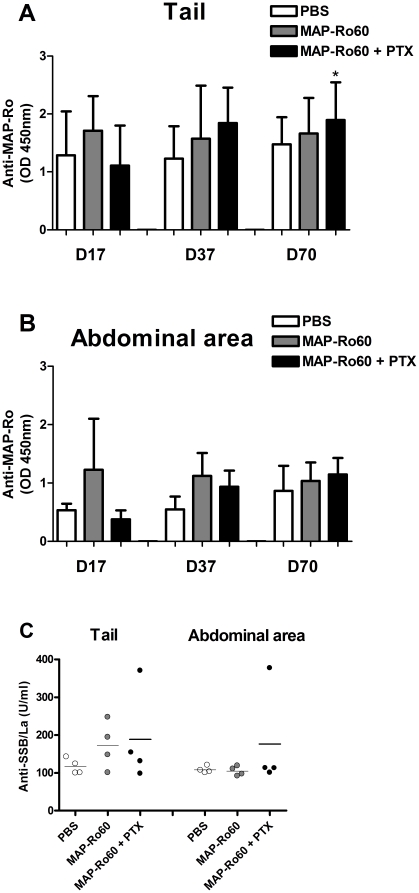
Induction of autoantibodies in SJL/J mice. Plasma from all animals used for saliva collection, were analyzed at the indicated time points for anti-MAP-Ro60 antibodies **(A-B)** or at the end of the study for anti-La antibodies (N = 4/group) **(C)**. The location of injection is indicated. Data shown are mean values +/- SD **(A-B)** or the mean value alone **(C)**. The P-value is indicated (* p<0.05) and was determined by unpaired Student's t-test.

Previous research demonstrated that Ro60 peptide immunization can trigger antibody formation against the whole 60-kDa Ro protein [14]. In contrast to the experience in Balb/c mice, MAP-Ro60 immunization of female SJL/J mice only resulted in a significant induction of antibodies compared with PBS immunized mice after the third boost in tailbase immunized + PTX mice (p = 0.03, Figure 2A). Furthermore, no difference in antibodies levels was observed in the mice immunized abdominally +/- PTX (Figure 2B).

It is not clear if the origin of anti-SSA/Ro and anti-SSB/La is linked, but epitope spreading after immunization has been reported [14]. Therefore we tested if immunization with Ro60 peptides could induce antibodies against the La antigen. In both tailbase and abdominal area immunized SJL/J mice, no increase in the mean titer of anti-SSB/La antibodies was detected (Figure 2C). However, 1 out of 4 mice clearly developed anti-La antibodies in MAP-Ro60 + PTX immunized mice at each injection site. These data show that MAP-Ro60 immunization does not lead to a more pronounced anti-MAP-Ro60 antibody production in SJL/J mice. Despite the lack of significant antibody formation in response to the immunization peptide, anti-La antibodies were detected in some of the Ro60 immunized mice indicating a low level of epitope spreading.

### No changes in focus score in MAP-Ro60 immunized SJL/J mice

**Figure 3:**
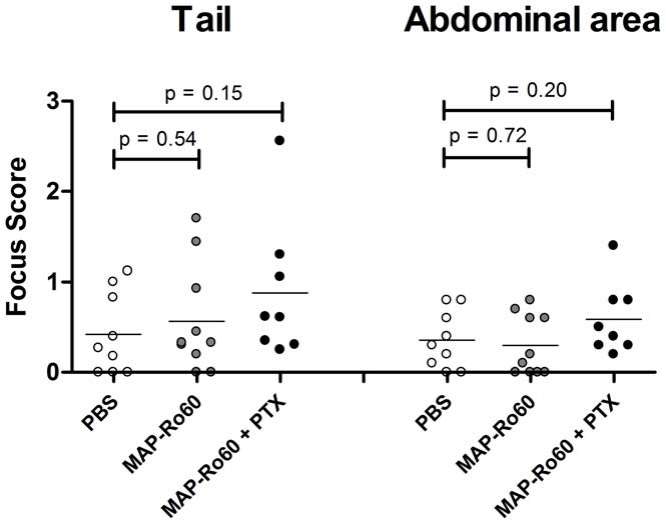
Unchanged salivary gland lymphocytic infiltrates in SJL/J mice. SGs from all animals used for saliva collection, were analyzed at the end of the study for lymphocytic infiltrates. The score of each mouse is shown individually. The location of injection is indicated. Data shown are mean values. P-values are indicated and were determined by unpaired Student's t-test.

SGs from immunized SJL/J mice were analyzed for lymphocytic infiltrates at the end of the study. In general, SJL/J mice showed low-grade local inflammation, as reflected by the focus score (average 0.39) at baseline. Although the addition of PTX to MAP-Ro60 immunized mice compared to PBS controls tended to increase the mean focus scores for the tailbase (0.88 versus 0.42, p = 0.15) and abdominal area (0.59 versus 0.36, p = 0.20) injection sites, this increase did not reach statistical significance (Figure 3). These data show that SJL mice are not more prone to the development of SG infiltrates after immunization. In addition, immunization with a modified Ro60 peptide, MAP-Ro60, does also not lead to an increased focus score. The use of an extra adjuvant, PTX, did not increase SG inflammation.

### Optimal decrease in salivary flow is dependent on the injection site and use of an adjuvant

**Figure 4:**
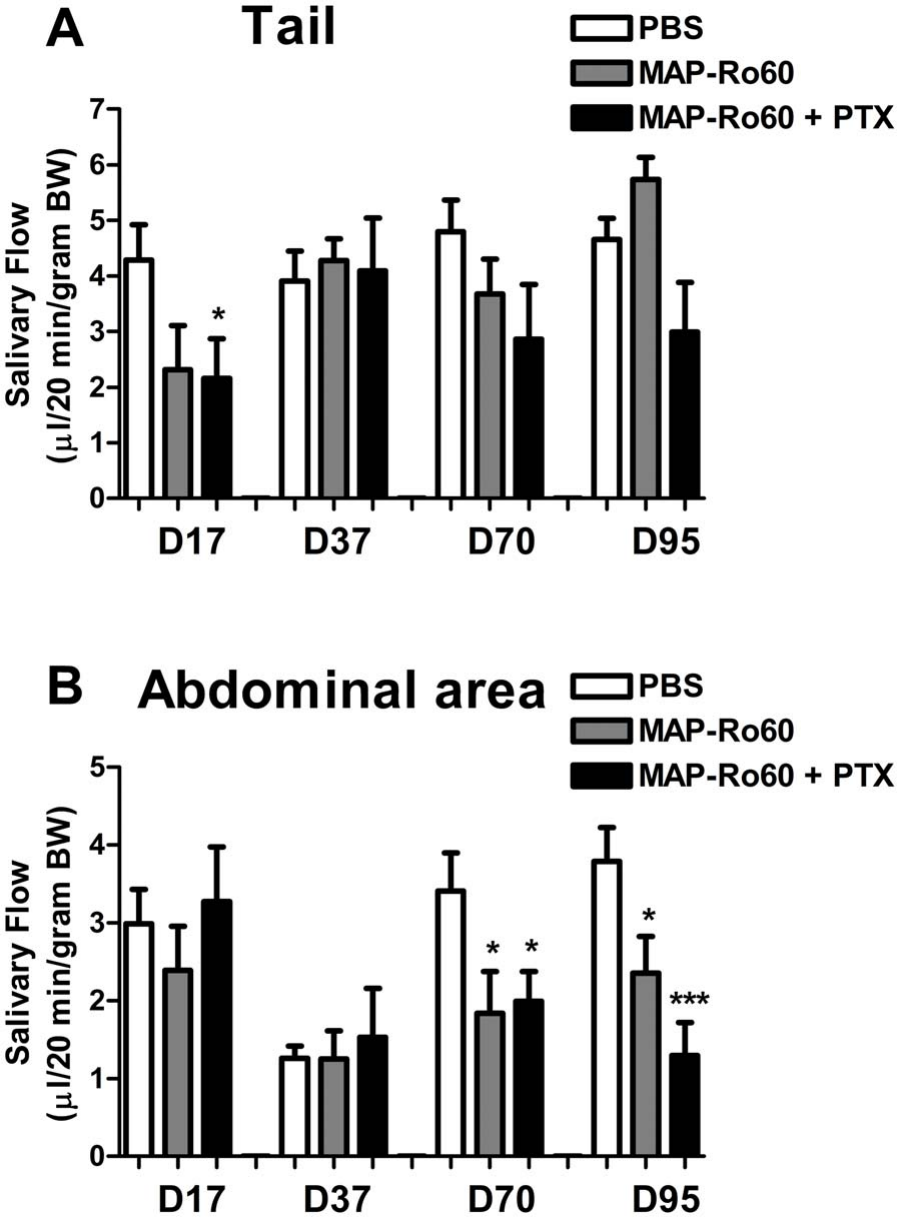
Salivary gland dysfunction in SJL/J immunized with MAP-Ro60 +/- PTX. SJL/J mice were immunized with MAP-Ro60 +/- PTX or PBS (mock) in the tailbase **(A)** or the abdominal area **(B)**. Data shown are mean values +/- SEM for 10 mice/group. Significant differences are indicated (*** p<0.0001; * p<0.05) and were determined by unpaired Student's t-test. D: number of days post primary injection.

Mice immunized in the tailbase showed a transient drop in SG function on day 17 that was only significant for the + PTX group (p = 0.04, Figure 4A). However, a sustained decrease in saliva production was seen when the abdominal area was immunized (p = 0.04 and p = 0.04 for day 70 and 95 respectively) and was more pronounced with the addition of PTX (p = 0.04 and p<0.001 for day 70 and 95 respectively, Figure 4B). Although, these data suggest that the presentation of the antigenic peptide (conventional versus MAP) or the use of SJL mice does not affect the time of onset of SG dysfunction following Ro60 peptide immunization, it does confirm the site of injection as being important in disease induction. Furthermore, the addition of PTX can increase SG impairment.

### MAP-Ro60 peptide increased IFN-γ producing lymphocytes

**Figure 5:**
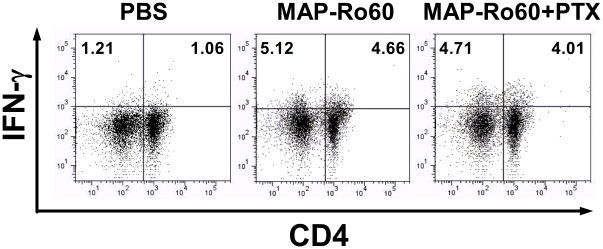
Increased IFN-γ releasing lymphocytes in salivary gland associated lymph nodes from SJL/J mice. The SG lymph nodes cells isolated from mice immunized with PBS (mock) or MAP-Ro60 +/- PTX were analyzed by flow cytometry assay for IFN-γ+ and specifically CD4+ IFN-γ+ (Th1) lymphocyte induction 10 days post primary immunization. The SG lymph node cells from one group (N = 10) are pooled and divided into 2 samples. Data shown is from one representative experiment.

Our study with Balb/c mice suggested that proinflammatory cytokines play a critical role in the loss of salivary gland activity in these mice. To further study if activated T cells were involved directly in the decreased SG function in the SJL mice, Th1 and Th17 populations were identified in isolated cells (day 10 post primary immunization) from associated lymph nodes (LNs) of the SGs. Around 1% IFN-γ+CD4+/- cells were found in control mice. In contrast, immunization with MAP-Ro60 +/- PTX increased IFN-γ+ cells (∼4-5% CD4+/- cells, Figure 5); no significant change in Th17 (CD4+IL-17+) was observed (data not shown). These data suggest that injection with MAP-Ro60 peptide promotes IFN-γ release from lymphocytes, localized in the SG LNs. This further confirms that the increase in IFN-γ might play a direct role in SG impairment.

## Discussion

The involvement of the Ro (SSA) antigen and anti-Ro antibodies in SS is shown in several studies [15], [16]. The majority of SS patients are seropositive for anti-Ro antibodies and these antibodies have been described to correlate with diseases activity [17]. These data suggest an important role for anti-Ro antibodies in SS, but the exact relevance of this antibody has not been elucidated yet.

In animal models, it has been shown that repetitive immunization with a Ro60 peptide or Ro60 protein can induce a mild SS-like disease, with reduced salivary flow and a low grade monocytic infiltration of the SG. Several controls in these studies, such as reverse and scrambled peptides as well as MAP backbone immunization, have established the specificity of the immune response to Ro^273–289^ [18]. However, the mechanism by which Ro60-immunization causes SS is still unknown. A direct debilitating effect of the autoantibodies is unlikely, since only several re-immunizations induce the onset of the disease and the presence of antibodies does not directly correlate with salivary gland dysfunction [5]. We therefore wanted to reproduce the effects of Ro60-immunization in Balb/c mice as previously described [5], and studied the cytokines that accompanied the SG dysfunction in order to get more insight into this phenomenon.

Ro60 immunized Balb/c mice developed autoantibodies to Ro60 peptide as expected. Over time, decreased salivary flow was observed as previously described [5]. When mice were immunized in the abdominal area as opposed to the tail base, more pronounced loss of function was observed. Recently, other investigators reported a similar injection site specific effects, in which flank injection with an autoantigen in an experimental autoimmune encephalomyelitis (EAE) mouse model was shown to be more effective in eliciting disease compared to footpad and tailbase injection [19].

Since the induction of antibodies was not significantly different between tailbase or abdominal area immunized Balb/c mice, this cannot be explained by a direct effect of these autoantibodies on SG dysfunction. Furthermore, SJL/J mice immunized with Ro60 peptide did not show anti-MAP-Ro60 antibodies or increased focus scores, again suggesting these changes do not directly account for the decreased SG function. Alternatively, induction of pro-inflammatory cytokines could be involved. Ro60-specific cells in the spleen of the abdominal area immunized Balb/c mice that developed xerostomia, produced consistently higher levels of IFN-γ and IL-12, compared with PBS or tailbase immunized mice. Also SG extracts of these mice contained higher levels of both IFN-γ and IL-12. This suggests that immunization in the abdominal area induces a more vigorous immune response than immunization at the tailbase, but the exact pathway remains to be elucidated. These data also emphasize the effect of IFN-γ on the salivary gland dysfunction in Ro60 peptide immunized Balb/c, and add to data that show the important role of IFN-γ on the growth and function of human SG cells [7], [8]. It also confirms the importance of IL-12 in SG dysfunction, as we have previously demonstrated in IL-12 transgenic mice [4].

It is further notable that the level of SG lymphocytic infiltrates did not correlate with changes in cytokines in the SG. Conceivably, systemic cytokines induced by Ro60-immunization circulate from the spleen and draining lymph nodes into the SG, or the epithelial cells within the SG serve as “lymphoid” cells and produce cytokines locally that later impair the function of the gland [20]. As shown for Balb/c mice, increased IFN-γ positive cells in the SG associated LNs of SJL/J following Ro60 peptide immunization also suggests the underlying mechanism of xerostomia is related to IFN-γ production. Overall, these data suggest that a Th1 cell mediated immune response with the secretion of cytokines plays a crucial role in the xerostomia observed in this model.

In conclusion, our data first show that reduced SG activity after repeated immunization is affected by the injection site: with the abdominal area injection leading to a more immunogenic and pathogenic effect than tailbase injections. The mechanism by which Ro60-immunization affects the salivary gland, appears to involve the induction of a Th1 cell response, emphasizing the crucial role of IFN-γ in SG dysfunction.

## Materials and Methods

### Animals

Female Balb/c and SJL/J mice (N = 80 and N = 90 respectively), 6–8 weeks old, were obtained from Jackson Laboratory (Bar Harbor, ME). Animals were housed in a pathogen-free facility. All procedures involving animals were performed in compliance with the National Institutes of Health (NIH) Guidelines on Use of Animals in Research. Animal protocols (#09-512) were approved by the National Institute of Dental and Craniofacial Research (NIDCR) Animal Care and Use Committee (ACUC) and the NIH Biosafety Committee.

### Peptides and immunization

Mice were immunized with Ro60 peptide as described earlier [21]. The following peptides (University of Oklahoma Health Sciences Molecular Biology core Facility, Oklahoma City, OK) were used in this study: Ro^273–289^ (NH2-*Leu-Gln-Glu-Met-Pro-Leu-Thr-Ala-Leu-Leu-Arg-Asn-Leu-Gly-Lys-Met-Thr*-COOH) for Balb/c immunization and MAP-Ro^273–289^ ((NH2-*Leu-Gln-Glu-Met-Pro-Leu-Thr-Ala-Leu-Leu-Arg-Asn-Leu-Gly-Lys-Met-Thr-Cys*)_8_-COOH) for SJL/J immunization and for detection of anti-Ro60 antibodies in ELISA (see below). A MAP is made up of a hepta lysine backbone upon which are built eight copies of the same peptide sequence (in this case 8 copies of Ro^273–289^). A MAP behaves like a low molecular weight protein. Briefly, on day 0 mice were immunized with 100 µg Ro60 peptide or phosphate buffered saline (PBS; Invitrogen, Carlsbad, CA) emulsified (1∶1) in complete Freund's adjuvant (CFA, Sigma-Aldrich, St. Louis, MO). Subsequently, animals were immunized with an equal amount of Ro60 peptide or PBS (1∶1) in incomplete Freund's adjuvant (IFA, Sigma-Aldrich, St. Louis, MO) on days 17, 37 and 70 post primary immunization (100 µl per animal) and sacrificed at day 95. Two subcutaneous (s.c.) injection sites were tested: the tailbase and the abdominal area. A separate group in each experiment of SJL/J mice was injected intraperitoneal (i.p.) with 0.1 µg/mouse PTX in addition to each peptide boost.

### Plasma preparation

For plasma preparation, blood was obtained via the retro-orbital plexus with a hematocrit tube (Drummond Scientific Company, Broomall, PA, USA) before each immunization. Plasma was separated by centrifugation at 2300xg for 5 min and stored at −80°C until further analysis.

### Determination of autoantibodies

Plasma samples were analyzed for autoantibodies against SSA/Ro and SSB/La. The enzyme-linked immunosorbant assay (ELISA) used to detect 60-kD MAP-Ro^273–289^ antibodies was described earlier [21]. The autoantibody against SSB/La (total Ig) was measured with a commercial available ELISA kit (Cat#5810, Alpha Diagnostic International, San Antonio, TX) according to the manufacturer's protocol.

### Saliva collection

Saliva was collected one day before each boost or sacrifice. Mice differ in their sensitivity to pilocarpine, and the dose needs to be optimized so that mice produce measurable amounts of saliva but do not over salivate and die due to plugging of the airways (personal observation). Optimal dosage of pilocarpine for Balb/c in our laboratory was set at 0.2 mg/kg bodyweight (BW) and 0.35 mg/kg BW for SJL/J mice. Saliva secretion was induced by s.c. injection of pilocarpine (Sigma-Aldrich, St. Louis, MO) and whole saliva was collected for 20 min from the oral cavity, and the volume was determined by weight.

### Histopathology

Submandibular glands (SMG) were removed for standard histological analysis using hematoxylin and eosin (H&E) staining from mice at the time of sacrifice. Scoring of histological sections was based on the focus score used in patients with SS [22]. Briefly, one of two whole submandibular glands were removed for histological analysis from at the time of sacrifice and placed O/N in 10% formaline. After fixation, the tissues were dehydrated in ethanol series and embedded in paraffin according standard techniques. 3 sections from the middle of the gland were cut at 5 µm and subsequently stained with hematoxylin and eosin (H&E). A focus is an aggregate of 50 or more lymphocytes and histiocytes per 4 mm^2^. Foci were counted through the whole section, in a total of three sections per salivary gland using a 40x magnification. The results were calculated and expressed as foci per 4 mm^2^. The focus scores were assessed blindly by two different examiners and the mean scores were determined.

### Detection of cytokines from cell cultures and SG homogenates

Splenocytes obtained from treated mice were isolated and cultured in 24-well plates at 5×10^6^ cells/mL RPMI-1640 medium (Invitrogen, Carlsbad, CA), containing HL-1 serum replacement (Cambrex Bioscience, Walkersville, MD), with or without 30 µg/mL Ro60 peptide. Supernatants were collected after 48 hr incubation.

Cytokine levels in the SG were determined after extraction of soluble protein from the SGs and correction for total protein concentration, which was determined with BCA™ protein assay kit (Pierce, Rockford, IL, USA). Cytokines were measured using a multiplex sandwich-ELISA assay (Aushon Biosystem, Billerica, MA). Duplicates for each sample were tested in three dilutions and the mean values of the duplicates from the optimal dilution were reported. Lower detection limits for this assay are: IL-4: 0.8 pg/ml, IL-10: 1.6 pg/ml, IL-17: 1.6 pg/ml, IFN-γ: 7.8 pg/ml.

### Flow cytometric identification of CD4+ IFN-**γ**+ lymphocytes

One million (10^6^) associated lymph node cells from the SGs (SG LN cells) were isolated and cultured in anti-CD3/anti-CD28 coated 24-well plate. Phorbol 12-myristate 13-acetate (PMA) and ionomycin (Sigma-Aldrich, St. Louis, MO) were added during the last 5 hr of total 48 hr incubation. Cells were harvested and stained with PerCP conjugated anti-mouse CD4 (BD Pharmingen, San Diego, CA) for 30 min on ice. Cells were fixed and permeabilized by a Fixation/Permeabilization kit (BD Pharmingen, San Diego, CA) and stained with allophycocyanin (APC)-conjugated anti-mouse IFN-γ (BD Pharmingen, San Diego, CA) according manufacturer**'**s protocol. Data were acquired using a FACSCalibur (BD Biosciences, San Diego, CA), and analyzed using Flowjo software (Tree Star Inc., Ashland, OR).

### Statistical analysis

One-way ANOVA and unpaired Student's t-test were used to compare differences between groups. All analyses were performed with GraphPad Prism statistical software (GraphPad Software Inc. version 5.01, La Jolla, CA) using a p value ≤0.05 as statistically significant.

## References

[1] Harley JB, Scofield RH, Reichlin M (1992). Anti-Ro in Sjogren**'**s syndrome and systemic lupus erythematosus.. Rheum Dis Clin North Am.

[2] Hansen A, Lipsky PE, Dorner T (2003). New concepts in the pathogenesis of Sjogren syndrome: many questions, fewer answers.. Curr Opin Rheumatol.

[3] Lodde BM, Mineshiba F, Kok MR, Wang J, Zheng C (2006). NOD mouse model for Sjogren**'**s syndrome: lack of longitudinal stability.. Oral Dis.

[4] Vosters JL, Landek-Salgado MA, Yin H, Swaim WD, Kimura H (2009). Interleukin-12 induces salivary gland dysfunction in transgenic mice, providing a new model of Sjogren's syndrome.. Arthritis Rheum.

[5] Scofield RH, Asfa S, Obeso D, Jonsson R, Kurien BT (2005). Immunization with short peptides from the 60-kDa Ro antigen recapitulates the serological and pathological findings as well as the salivary gland dysfunction of Sjogren**'**s syndrome.. J Immunol.

[6] Bombardieri M, Barone F, Pittoni V, Alessandri C, Conigliaro P (2004). Increased circulating levels and salivary gland expression of interleukin-18 in patients with Sjogren**'**s syndrome: relationship with autoantibody production and lymphoid organization of the periductal inflammatory infiltrate.. Arthritis Res Ther.

[7] Meehan S, Wu AJ, Kang EC, Sakai T, Ambudkar IS (1997). Interferon-gamma induces a decrease in the intracellular calcium pump in a human salivary gland cell line.. Am J Physiol.

[8] Wu AJ, Chen ZJ, Kan EC, Baum BJ (1997). Interferon-gamma-induced JAK2 and STAT1 signalling in a human salivary gland cell line.. J Cell Physiol.

[9] Papenfuss TL, Rogers CJ, Gienapp I, Yurrita M, McClain M (2004). Sex differences in experimental autoimmune encephalomyelitis in multiple murine strains.. J Neuroimmunol.

[10] Sasaki M, Allina J, Odin JA, Thung SN, Coppel R (2002). Autoimmune cholangitis in the SJL/J mouse is antigen non-specific.. Dev Immunol.

[11] Silver PB, Chan CC, Wiggert B, Caspi RR (1999). The requirement for pertussis to induce EAU is strain-dependent: B10.RIII, but not B10.A mice, develop EAU and Th1 responses to IRBP without pertussis treatment.. Invest Ophthalmol Vis Sci.

[12] Cruz LJ, Iglesias E, Aguilar JC, Gonzalez LJ, Reyes O (2004). A comparative study of different presentation strategies for an HIV peptide immunogen.. Bioconjug Chem.

[13] Oscherwitz J, Yu F, Cease KB (2009). A heterologous helper T-cell epitope enhances the immunogenicity of a multiple-antigenic-peptide vaccine targeting the cryptic loop-neutralizing determinant of Bacillus anthracis protective antigen.. Infect Immun.

[14] Scofield RH, Kaufman KM, Baber U, James JA, Harley JB (1999). Immunization of mice with human 60-kd Ro peptides results in epitope spreading if the peptides are highly homologous between human and mouse.. Arthritis Rheum.

[15] Abelson AK, Delgado-Vega AM, Kozyrev SV, Sanchez E, Velazquez-Cruz R (2009). STAT4 associates with systemic lupus erythematosus through two independent effects that correlate with gene expression and act additively with IRF5 to increase risk.. Ann Rheum Dis.

[16] Rhodes DA, Ihrke G, Reinicke AT, Malcherek G, Towey M (2002). The 52 000 MW Ro/SS-A autoantigen in Sjogren**'**s syndrome/systemic lupus erythematosus (Ro52) is an interferon-gamma inducible tripartite motif protein associated with membrane proximal structures.. Immunology.

[17] Praprotnik S, Bozic B, Kveder T, Rozman B (1999). Fluctuation of anti-Ro/SS-A antibody levels in patients with systemic lupus erythematosus and Sjogren**'**s syndrome: a prospective study.. Clin Exp Rheumatol.

[18] James JA, Gross T, Scofield RH, Harley JB (1995). Immunoglobulin epitope spreading and autoimmune disease after peptide immunization: Sm B/B**'**-derived PPPGMRPP and PPPGIRGP induce spliceosome autoimmunity.. J Exp Med.

[19] Pastor S, Minguela A, Mi W, Ward ES (2009). Autoantigen immunization at different sites reveals a role for anti-inflammatory effects of IFN-gamma in regulating susceptibility to experimental autoimmune encephalomyelitis.. J Immunol.

[20] Fox RI (2005). Sjogren's syndrome.. Lancet.

[21] Scofield RH, Henry WE, Kurien BT, James JA, Harley JB (1996). Immunization with short peptides from the sequence of the systemic lupus erythematosus-associated 60-kDa Ro autoantigen results in anti-Ro ribonucleoprotein autoimmunity.. J Immunol.

[22] Vivino FB, Gala I, Hermann GA (2002). Change in final diagnosis on second evaluation of labial minor salivary gland biopsies.. J Rheumatol.

